# Population Recruitment Strategies in the Age of Bots: Insights from the What Is on Your Plate Study

**DOI:** 10.1016/j.cdnut.2025.107442

**Published:** 2025-04-15

**Authors:** Emily G Elenio, Alison Tovar, John San Soucie, Maya K Vadiveloo

**Affiliations:** 1 Department of Behavioral and Social Sciences, School of Public Health, Brown University, Providence, RI, United States; 2 Applied Ocean Physics and Engineering Department, Woods Hole Oceanographic Institution, Woods Hole, MA, United States; 3 Department of Mechanical Engineering, Massachusetts Institute of Technology, Cambridge, MA, United States; 4 Department of Nutrition, College of Health Sciences, The University of Rhode Island, Kingston, RI, United States

**Keywords:** web-based food frequency questionnaire, dietary assessment, Supplemental Nutrition Assistance Program, bots, online survey security, fraud prevention, data integrity

## Abstract

**Background:**

To evaluate state-wide nutrition policies, valid tools are required to gather sufficient sample sizes. Remote data collection, including web-based dietary assessments, offers convenience for participants and researchers and enables faster and more diverse recruitment. However, it presents challenges, including risk of bots compromising data integrity.

**Objectives:**

This study describes the technical survey design of an ongoing longitudinal study, which is evaluating a state-wide Supplemental Nutrition Assistance Program (SNAP) incentive program, discusses strategies to prevent and identify bots, duplicates, fraudulent entries, and implausible data, and provides recommendations to improve future public health nutrition research.

**Methods:**

From May to September 2023, SNAP participants from Rhode Island and Connecticut were recruited to complete an online food frequency questionnaire (FFQ) and a demographic survey. Given the large sample and online format, our interdisciplinary team designed the technical backend to optimize participants’ convenience while ensuring data quality through an automated system that assessed FFQ responses. To prevent bots and duplicates, we created duplicate application programming interfaces (API), randomly called participants, and evaluated Completely Automated Public Turing Test to Tell Computers and Humans Apart (reCAPTCHA), geotags, and Internet Protocol (IP) addresses.

**Results:**

Using a combination of text blasts and in-person recruitment, we enrolled 1367 participants, with text blasts proving the most effective strategy (∼60% of participants). Midway through recruitment, we identified 544 potential bots that completed the screener, with duplicate IP addresses and geotags from outside the recruitment area serving as strong indicators of bot activity. At baseline, 112 participants failed FFQ data quality checks, prompting follow-up by research assistants. Our automated duplicate and FFQ APIs saved countless hours of staff time.

**Conclusions:**

Remote data collection tools were critical for meeting recruitment goals and ensuring our data authenticity. A combination of strategies is necessary to effectively mitigate against bots and ensure plausible responses. Widely available, built-in tools (e.g., reCAPTCHA) are helpful but are insufficient alone. Customized solutions like our automated systems may be critical for future researchers to maintain data integrity.

## Introduction

Remote data collection, including online or web-based surveys, is increasingly popular for health studies [1]. Remote data collection, which became necessary during the COVID-19 pandemic, has continued to rapidly accelerate [2,3]. Remote data collection can be advantageous as it enables more rapid recruitment across geographies [2,4]. Furthermore, remote data collection can be low-cost, reach hard-to-recruit populations and offer greater convenience for participants and researchers, often ensuring suitable sample sizes [[3], [4], [5], [6], [7]]. For example, within nutrition research, traditional paper-based diet assessment methods are time-consuming and burdensome. Emerging computer-administered food frequency questionnaires (FFQ) offer some benefits over traditional paper-based forms, including greater support for complex skip patterns, reduced error introduced through manual data entry into analysis software, and lower costs [[7], [8], [9]]. These benefits support participant convenience by providing a more personalized experience through complex skip patterns while enabling faster data collection and analysis for researchers. Despite the benefits of remote data collection tools, research suggests they are not a panacea for improving recruitment and retention in the post-COVID-19 era [3,4,10].

Certain limitations of remote data collection methods have become increasingly evident. For example, people from lower socioeconomic backgrounds, who are often at higher risk of disease, experience barriers to accessing online surveys due to low technology literacy [3,11]. Furthermore, as remote data collection expands, new risks emerge that raise concerns about participant trust and data integrity due to the presence of automated survey-taking scripts, often referred to as bots, and fraudulent actors [3,5,6,12,13]. Although previous research has documented some best practices on how to protect data, the effectiveness and feasibility of such strategies are underdeveloped [14]. For example, prior studies have documented standard approaches to preventing bots, including using the Completely Automated Public Turing Test to Tell Computers and Humans Apart (reCAPTCHA), removing responses with implausible response times, and assessing open-ended answers for nonsensical responses [6,13,15,16]. However, few studies have been conducted within the field of public health, and most literature focuses on post hoc analyses to identify bots [2,6]. Limited research on the effectiveness of dynamically adapting security measures to prevent bots throughout the research process holds particular importance in public health. This gap is critical, as effective security measures are essential to ensure data integrity in public health studies, where findings directly impact population health interventions and policy. However, these measures can also pose challenges: they may inadvertently increase participants’ mistrust and deter those with low digital literacy from participating, potentially skewing the representation of high-risk populations in public health research.

The goal of this article is to describe the technical survey design of the “What’s On Your Plate” study and discuss various strategies, including computer science techniques, used to prevent and identify bots, duplicates, and fraudulent responses. In our study, we implemented security features based on a priori knowledge and continued to adapt them in response to incoming data, ensuring that the core of the study remained unaffected. Given our focus on recruiting participants from low socioeconomic backgrounds, we carefully balanced security checks to avoid adding participant burden that could increase mistrust and frustration. Finally, through an examination of our survey infrastructure, we highlight the unique challenges and considerations involved in conducting public health nutrition research.

## Methods

The “What’s On Your Plate” study uses a longitudinal differences-in-differences design to evaluate changes in fruit and vegetable intake and diet quality to evaluate a state-wide Supplemental Nutrition Assistance Program (SNAP) incentive program. The methods describing this study have been published elsewhere but are briefly described below [17]. Participants were recruited from Rhode Island (RI) (intervention site) and Connecticut (CT), which served as the control state. Baseline data were collected from May through September 2023 and 1367 participants ultimately enrolled and completed the baseline survey. Follow-up data collection spanned from May 2024 through September 2024 with a total of 837 participants who completed follow-up. At study onset, it was determined that online data collection would be necessary to reach a racially and ethnically diverse population with low-income backgrounds across state lines and within a relatively short time frame. The survey contained 2 parts: a self-administered FFQ which was collected using VioScreen and a demographic survey which was coded into Qualtrics, a well-established survey platform [7,18]. All participants who completed the survey and met data quality checks, which are described in greater detail below, received a $50 e-gift card. This research was approved by the Institutional Review Boards (IRB) of the University of Rhode Island and Brown University. Participant consent forms delineated how e-gift cards would be sent after a data quality check; if the survey data did not meet the criteria, the participants would be contacted to clarify their answers.

The survey design and study goals introduced many challenges, including risk of bots, duplicates, and fraudulent responses, and building security measures was challenged by participants’ limited technology literacy. Mitigating the aforementioned challenges was difficult as the research community only recently pivoted to online data collection measures, and there are no authoritative guidelines or best practices for preventing bots. To successfully recruit the sample and rigorously collect data, we created a system that balanced addressing security risks while maintaining ease and accessibility to participants.

### Recruitment methods

**FIGURE 1:**
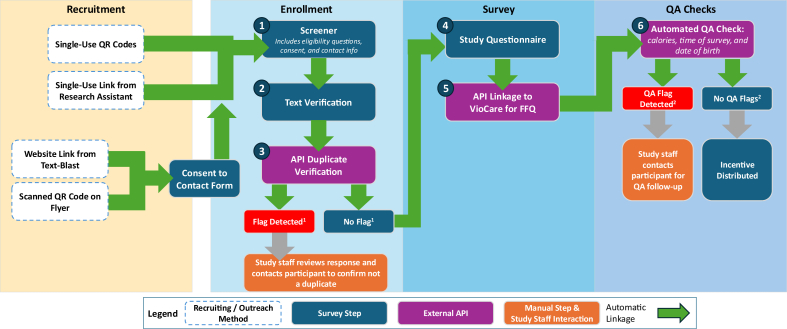
Baseline survey structure. ^1^Duplicate flag as detected by Qualtrics or duplicate email or phone detected by the API. ^2^Flag triggered if age provided in screener did not match date of birth in full survey and/or if participant completed food frequency questionnaire in <10 min, or if participant reported < 600 kcals or > 10,000 kcal. APIs, application programming interfaces; AWS, Amazon Web Services; DOB, date of birth; FFQ, food frequency questionnaire; QA, quality assurance; QR, quick response.

Given the large sample size required across 2 states, we employed a multipronged recruitment strategy. Participants were recruited using various strategies which are depicted in Figure 1. Research assistants recruited participants at community events such as blood drives and food pantry events in RI and CT. Text blasts were sent to the Special Supplemental Nutrition Program for Women, Infants, and Children (WIC) listservs. Additionally, community partners helped support recruitment and provided feedback on the survey instruments and recruitment process. Community partners provided feedback that households from low socioeconomic backgrounds often access resources from multiple community organizations, which can lead to some individuals being inadvertently exposed to recruitment efforts at >1 site. This feedback was valuable as it helped inform the survey structure and security during the early phases.

Community partners were given packets that detailed the study overview and included brief scripts for staff members to use while recruiting. To facilitate recruitment, community partners were given flashcards with single-use quick response (QR) codes and flyers with a QR code that led to a “consent to contact” link. The QR code on the flyer was reusable so community partners could circulate via limited email distributions. If participants chose to complete the consent to contact link and provided their contact information, the study team sent the interested participant a unique screener link that was automatically generated by Qualtrics.

### Survey structure

Based on feedback from community partners, a review of Qualtrics security features, and literature speaking to risk of bots, we designed a baseline survey structure that could identify potential duplicates and bots, without significantly compromising speed or participant convenience. Our system needed to reliably identify duplicates and bots without impeding participation from “true” participants. To achieve this, we created application programming interfaces (APIs) which are sets of programmed instructions that dictate how applications (e.g., Qualtrics) interact with other applications [19]. For instance, an API was used to transfer participants’ data from the FFQ platform to Qualtrics. All APIs were created using Amazon Web Services (AWS), a comprehensive cloud programming platform that is commonly used for API development [20]. We used APIs and built-in Qualtrics features, such as workflows which automatically manipulate data within Qualtrics and transfer data to APIs, to seamlessly pass data between the different components of the surveys [21]. Examples of Workflow functions are discussed below. Qualtrics refers to information that is passed through surveys as “embedded data.” We consulted with graduate-level computer science research assistants to support the development, testing, and application of our APIs. At baseline, text verification was included due to the various recruitment strategies and risk of bots. However, at follow-up, the text verification step was removed and replaced with a grocery store receipt verification to increase confidence that participants were currently receiving SNAP benefits.

For baseline data collection, we programmed the screener, text verification question, and survey questionnaire into Qualtrics and created 3 different surveys which were linked using XMDirectories, which are Qualtrics’ “contact lists” that house prepopulated data and/or select survey data [22], and workflows. Separating the components into distinct parts was necessary to support various security features and facilitate the APIs. The survey flow and features are depicted in Figure 1. Participants accessed the screener using single-use QR codes or custom invite links. The eligibility criteria were as follows: participants must be 18 or older, speak English or Spanish, receive SNAP benefits, live in RI or CT, and have access to an email address and a phone that receives text messages. If the participant met the eligibility criteria and provided consent and their contact information, they proceeded to text verification which was triggered by a Qualtrics Workflow that sent a unique 6-digit code to their cell phone number. After the screener was submitted, the participant moved to the text verification survey which used a prepopulated XMDirectory with preset passcodes. After text verification, an API determined if the participant entry was a duplicate as identified by email or phone number. If no duplicate flags were generated by the API, a Qualtrics JavaScript Object Notation (JSON) event was triggered, and the participant was sent an email invitation to the baseline survey. To ensure that embedded data were passed across surveys and to support authentication, the survey began in Qualtrics, and then an API connected participants to VioScreen for the FFQ; afterward, participants were linked back to Qualtrics to complete the sociodemographic survey. Other APIs were used to transfer data, such as reported calories, start time, end time, and age, from VioScreen back to Qualtrics. Aside from embedded data that were passed through the APIs, data from Qualtrics and VioScreen were stored separately.

**FIGURE 2:**
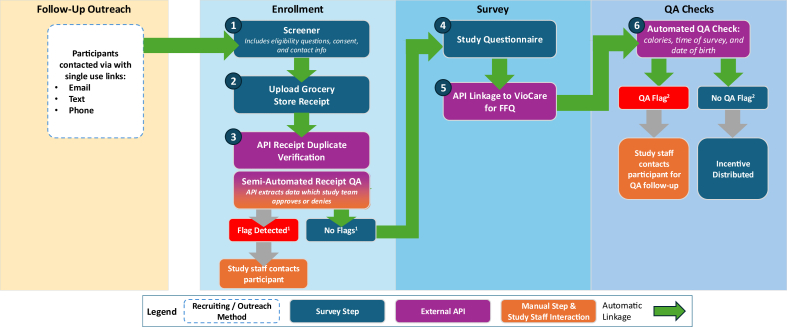
Follow-up survey structure. ^1^Receipt flag if receipt does not show EBT card was used, receipt older than 30 days from submission date, or receipt is a duplicate (duplicate photo or EBT card number). ^2^QA flag triggered if age provided in screener did not match date of birth in full survey and/or if participant completed food frequency questionnaire in < 10 min, or if participant reported < 600 kcals or > 10,000 kcal. APIs, application programming interfaces; EBT, electronic benefit transfer; FFQ, food frequency questionnaire; QA, quality assurance.

The follow-up survey used a very similar structure to the baseline, except text verification was replaced with a receipt verification step and the contact list was limited to the participants who were eligible for follow-up (Figure 2). Participants’ contact information was uploaded to an XMDirectory in batches. Because all links were authenticated and limited to the XMDirectory, no duplicate API was used. After completing the screener and providing consent, participants were asked to upload a picture of a grocery store receipt within the past 30 days that showed that their electronic benefit transfer (EBT) card had been used. After submission, the receipt was sent via an API to an AWS Lambda function, which is a serverless cloud-based program that runs events when triggered by an external command [23], that assessed if the photo was a duplicate and extracted data points using Amazon’s Textract, a machine learning tool that extracts text from images [24].

### Security features: authentication

Safeguards were built to prevent duplicates along with bots. All surveys were authenticated using Qualtrics built-in features and XMDirectories. Random alphanumeric “passcodes” and 6-digit “secret codes” were generated in Excel for each ID and then uploaded to an XMDirectory. Each Qualtrics link was customized and then authenticated using the participant’s study ID and the passcode or “secret code.” Two different unique codes, passcode and secret code, were used so participants or bots could not use the “secret code” of the screener on the baseline survey. The structure of the authenticated links is shown in Supplemental Figure 1. New passcodes and secret codes were generated for follow-up outreach. Participants accessed their customized links by scanning QR codes at baseline recruiting or via email at follow-up. Qualtrics authenticated each participant by matching their study ID and passcode or secret code from the link to the information stored in the respective XMDirectory.

### Security features: duplicates

To facilitate recruitment, identification and mitigation strategies for possible duplicates needed to be efficient. Prior to launch, we considered using Qualtrics built-in security features, including RelevantID, which assigns a duplicate score between 0 and 100, with scores above 75 likely indicating a duplicate [25]. Although RelevantID itself does not prevent duplicates, it assigns scores to 4 different subcomponents to help identify them [25]. Given the limitations, we determined that using RelevantID was inappropriate in some instances. For in-person recruitment, it was inefficient for research staff to investigate each duplicate flag—particularly because Qualtrics does not identify what aspect was duplicative and the associated match. Additionally, participants recruited on-site at community partner organizations were at higher risk of being marked as duplicates if they shared the same Wi-Fi network. Initially, we captured RelevantIDs and found that many participants were incorrectly flagged as duplicates, so for in-person recruiting periods, we did not use RelevantID. However, for large text blasts where some of the above in-person issues were not relevant, we did use RelevantID given the greater risk of bots.

To ensure comprehensive detection of duplications, we created our own API where after a participant completed text verification their name, subject ID, email, and phone number were sent to AWS using an AWS Lambda. All sent data were stored in an Amazon DynamoDB table, which is Amazon’s serverless database product [26]. If a duplicate was detected, the participant was notified via email that there was an issue with their response, an email was sent to the entire research team, and the duplicate ID was automatically added to an Excel sheet for tracking. Research assistants followed up with all duplicate IDs and informed them they could only take the survey once. As community partner feedback indicated that multigenerational households or spouses may share a phone or email, we built in an override system. For example, on a few occasions, a participant submitted the same email or phone number as another participant; if the research staff was able to confirm that the 2 people were distinct, they instructed the participant to provide another phone number and/or create an email address. Only the study coordinator was able to submit override requests which added the participant to the survey XMDirectory in Qualtrics. At baseline, Internet Protocol (IP) addresses were monitored after large text blasts to WIC listservs. Duplicate IP addresses were flagged, and research assistants followed up with participants to confirm that they were unique individuals and not bots.

At follow-up, the duplicate API was not used because there was a very limited risk of repeated entries. Rather, undetected duplicates from the baseline posed the greatest risk at follow-up. Duplicate detection at follow-up included identifying IP addresses and EBT card numbers which were passed as part of the receipt verification using an API and saved in a DynamoDB table. All possible duplicate participants were called and asked to confirm their information including date of birth, full name, zip code, and the last 4 digits of their EBT number.

### Security features: bots

At the start of baseline fielding, risk of bots was minimal as most recruiting was conducted by community partners and research assistants at community events. Because of the low response rates from in-person recruiting, 3 text blasts were sent to WIC listservs in RI and CT midway through baseline recruitment. In 2023, there were an estimated 17,235 and 47,847 people receiving WIC in RI and CT, respectively [27]; as a result, there was a greater risk of bots given the sizable sample.

For text blasts, participants first completed a “consent to contact” form in Qualtrics where they provided their contact information and then a screener link was automatically sent to them. Given the high volume and risk of bots, consent to contact responses with suspicious data did not receive the screener link. To detect potential bots, Qualtrics built-in features were used. Responses were flagged and prevented from automatically receiving a screener link if the RelevantID Fraud Score was 30 or greater and/or if the reCAPTCHA score was 0.5 or greater [25]. After analyzing the responses from the first text blast and discussing with the research team, we implemented a flagging system for the second and third text blasts: responses were flagged if their geolocation was outside of RI, CT, and neighboring states.

During high-volume periods such as the text blasts, participants who passed the screener were randomly called to ensure they were not bots. Research assistants called approximately 1 participant for every 10 responses and asked the participant to confirm their information from the screener including their name, email, zip code, and date of birth. Randomly selected participants could not complete the survey and receive their compensation without confirming their identity with a research assistant.

### Data quality checks

**FIGURE 3:**
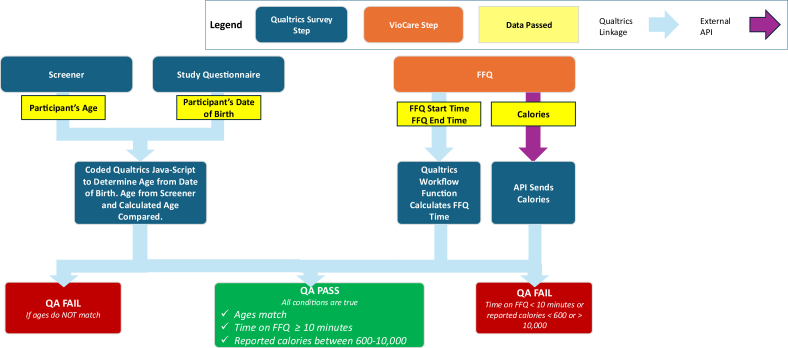
Quality assurance data check process and structure. APIs, application programming interfaces; FFQ, food frequency questionnaire; QA, quality assurance.

Because we utilized an online FFQ, data quality checks were necessary to ensure data rigor and served as an additional security measure against bots. Figure 3 shows the backend structure of the data quality checks and criteria. The protocol for the FFQ data quality checks was informed by a priori criteria from the validation study; response times that were under 10 min, with fewer than 600 daily calories reported, and/or with greater than 10,000 daily calories reported were considered invalid [7,18]. Another API was used to transfer data from VioScreen and assess if the FFQ data met criteria. For all invalid responses, the research team was notified, and the participant was emailed asking them to reach out to set up a time to speak with a study staff member. If participants did not respond to the first email, research assistants made at least 3 attempts to contact the participant via email, phone call, and/or text message. Once the participant was contacted, they were asked to set up a time for a researcher to readminister part or all of the FFQ with the participant via Zoom or a phone call. Research assistants underwent training with the co-PI and/or more senior research assistants on how to readminister the FFQ via the phone or Zoom. These data checks prevented rapid response times observed by some bots and ensured the quality of the data.

In addition to the FFQ, logic data checks were built into the surveys. Participants were asked to provide their age in the screener and their date of birth in the full survey. At baseline, a JavaScript function was built into Qualtrics that calculated the participants' age based on their date of birth. Responses were flagged as invalid if the difference between the reported age in the screener and the calculated date of birth was greater than one year. At follow-up, participants were flagged as “FAIL” if their date of birth did not match the date of birth reported at baseline.

## Results

### Recruitment strategy outcomes

At baseline, we exceeded our recruiting goal of 1250 and ultimately enrolled 1367 participants. Table 1 describes the source of participants from each recruiting strategy.

**TABLE 1:** Source of participants at baseline.

Source	*N* (%)
Community partners	205 (15.0)
Research assistants	111 (8.1)
Text blasts	810 (59.2)
Flyers	241 (17.6)

The overwhelming majority of participants were recruited via text blasts.

#### Bot analysis

At baseline, the flyer link was sent to a suspected bot email. Within 12 hours of the suspected breach, the flyer link was disabled limiting participation to the single-use paper QR codes or through a research assistant. All new entries after the breach were reviewed. During the approximately 8 hours between the breach and the link shutdown, 554 likely bots accessed the screener, and initially it appeared that 51 completed the survey. Study staff called all 51 participants who took the full survey during this time; of them, only 1 was deemed to be a real participant as they confirmed their full name, date of birth, and zip code during a phone call. The calls to all other expected bots were disconnected phone numbers. To improve the security of the survey for the text blasts, metadata from the consent to contact form was analyzed. At this point in fielding, most respondents were recruited in-person by community partners and/or research assistants. A total of 1291 responses were submitted to the consent to contact form from study launch through the bot breach, of those 523 were identified as likely bots using metadata indicators which are shown in TABLE 2, TABLE 3 and by phone calls.

**TABLE 2:** Comparison of metadata^1^.

	Suspected bots(*n* = 523)	True participants(*n* = 768)
	*N* (%)	*N* (%)
Duplicate IPs	404 (77.2)	262 (34.1)
reCAPTCHA fails	217 (41.5)	0 (0)

Abbreviations; IP, Internet Protocol; reCAPTCHA, Completely Automated Public Turing Test to Tell Computers and Humans Apart.

1 Using Qualtrics predefined threshold of < 0.5.

**TABLE 3:** Comparison of geotags^1^.

	Suspected bots(*n* = 523)	True participants(*n* = 214)^1^
	*N* (%)	*N* (%)
Geotags within CT, RI, MA, and NY	151 (28.9)	175 (81.8)

Abbreviations: CT, Connecticut; MA, Massachusetts; NY, New York; RI, Rhode Island.

1 Reflects participants who had geotag data.

Of the indicators measured, reCAPTCHA failures proved to be the most specific as none of the true participants triggered a reCAPTCHA flag. However, this method only identified approximately 41.5% of bots. The majority of suspected bots used duplicate IP addresses, yet about one-third of true participants were also flagged for duplicate IPs. As bots breached the consent to contact form, which then led to the full screener, participants and bots could take the consent to contact form multiple times. Research assistants called participants who took the consent to contact form multiple times and confirmed their identity. Often, true participants took the consent to contact form multiple times erroneously. For suspected bots, phone numbers were often not functional (e.g., disconnected number, dial tone, or failed Google voice subscribers).

Geotags were also assessed. At the start of the study, geotags were not collected so initially the first 554 true participants to complete the consent to contact form did not have geotag data. Only 28% of suspected bots had geotags from within the target recruiting area, defined as CT and RI along with Massachusetts and New York, compared with 82% of true participants. Of true participants, New Jersey was the most common geotag outside of the target region (*n* = 15), whereas Michigan was the most common among suspected bots (*n* = 79). There were a total of 30 unique states and/or countries detected among suspected bots compared with 15 of true participants.

#### Data quality checks

At baseline, over 200 participants failed one of the data quality checks, of those 112 were for the FFQ. Research assistants completed calls with all 112 participants and re-reviewed their responses.

#### Summary of results

In Table 4 [2,6,[12], [13], [14],^16^,^25^,^28^,^29^], we present a summary of the different strategies that were used with implications for future research. Overall, the automated data quality checks for the FFQ were the most influential to supporting scalability of the study, whereas the automated APIs coupled with built-in Qualtrics features were critical to identifying bots and fraudulent actors. Random phone calls and duplicate IP checking offered greater confidence and security.

**TABLE 4:** Summary of security measures, description, effectiveness, and implications for other research^1^.

Type of check	Strategy	Description and rationale	Effectiveness in our study	Implications for other studies
Bot and duplicate checks	reCAPTCHA	● Built-in Qualtrics function that assigns a score and is intended to detect bots [14,25,28] ● Standard and frequently used for bot detection [2,6,12,13,16]	● Extremely specific (0% of “true” participants flagged) ● See Table 2	● Low cost and standard function ● Given limited sensitivity, should be used with other measures
	Duplicate IPs	● Detection of the same IP address across multiple participants ● Can detect potential bots or duplicates [29]	● Effective at identifying bots with a high false-positive rate ● See Table 2	● Requires survey infrastructure that can detect IPs ● Not suitable for studies with participants from the same household/organization
	RelevantID	● Built-in Qualtrics feature that assigns a score on likelihood of a duplicate response [25] ● See Methods—Security Features: Duplicates Section	● Effective during text blasts for identifying bots, but limited utility when recruiting at community partners ● Key limitation is Qualtrics only identifies the “second” duplicate	● Similar limitations to duplicate IP, especially when recruiting in-person where Wi-Fi is available and/or when multiple members of the same household are participating
	Duplicate API	● Customized API tool built-in AWS to detect duplicate emails and/or phones ● See Methods—Security Features: Duplicates Section	● Extremely effective at identifying duplicates ● Throughout survey, no issues with duplicate API and served as an efficient tool for identifying duplicate screener responses	● Requires knowledge of AWS or similar tool for API development ● Highly customizable and flexible tool ● Requires extensive testing prior to fielding
Data quality and bot checks	Random phone calls	● Calls to participant to confirm their basic information	● Extremely effective but resource intensive	● Impractical to call every potential participant in a large-scale study ● Requires ongoing efforts to respond to return phone calls ● Offers the greatest level of confidence
	Nutrition API data quality check	● API that extracted data from VioCare to assess number of calories and time spent on the survey ● API automatically generated a “PASS” or “FAIL” rating for each participant; “PASS” were sent gift cards and “FAIL” participants were asked to set up a time with research assistants ● See Methods—Data Quality Checks section	● API allowed for rapid assessment of FFQ data which greatly reduced resourcing needs ● Approximately 8% of participants triggered FFQ flag	● Automated review of FFQ data significantly improves efficacy which can support greater sample sizes ● Comprehensive testing is required, prior to launchr ● Recommend ongoing monitoring to assess thresholds as certain populations may warrant different cutoffs

Abbreviations: API, Application Programming Interface; AWS, Amazon Web Services; FFQ, Food Frequency Questionnaire; IP, Internet Protocol; reCAPTCHA, Completely Automated Public Turing Test to Tell Computers and Humans Apart.

## Discussion

We found through a combination of customized APIs, data quality checks, and participant verification that we were able to successfully recruit and collect in-depth data from 1367 "true" participants over a period of 4 months. There is a critical need for public health nutrition researchers to ensure rigorous data collection. Although risk of bots cannot be understated, we believe researchers should consider the immense advantages of online surveys and pursue partnerships with computer science experts to build more secure survey infrastructures. Through close collaborations with computer science experts, we were able to customize our survey, build in security features, and integrate Qualtrics, APIs, and the FFQ, while keeping the user experience in mind. Furthermore, reported recruitment strategies in nutrition and physical activity research are often vague and details regarding implementation and yield rates are rarely reported [30]. Our methods and results presented in this article provide detailed information on effective recruiting tactics, provide characteristics of bot-like responses, detail potential prevention, and data quality measures, and discuss how methodologies can evolve to better prevent bots while fielding an online survey, particularly within public health nutrition.

Despite the success of this data collection strategy, some may gravitate toward traditional recruitment methods and continue to view online recruitment as suboptimal or view the application of these tools as daunting. However, it is important to underscore that text blasts were essential to achieving our recruiting target in our desired timeline. Traditional recruitment methods, including partnerships with community organizations and direct in-person recruitment, were not viable strategies to recruit a large sample across 2 states for this pilot study, and text blasts played a pivotal role in reaching the target sample size efficiently. Without thoughtful design for security, text-based recruitment could have easily been compromised by fake participants or bots, underscoring the need for intensive security measures. Although implementing the text blasts required significant resources and the development of automated systems, this approach yielded the highest number of participants. By using multiple APIs and automated systems, a single research assistant was able to manage participant questions and inquiries despite 20,000–40,000 potential participants. The APIs effectively identified and held duplicates and high-risk responses, minimizing the need for manual review which saved hours of research assistant time and thus cost. Excluding randomized control trials, few public health studies report their yield rates of recruitment tactics [31]. A 2009–2011 study investigating the impact of physical activity messaging recruited 599 males from RI and North Carolina over 19 months; mass mailings (over 340,000 pieces), and email blasts (over 25,000 emails) were the most effective recruitment strategies [31]. Conversely, a Delphi panel and systematic review of nutrition and physical activity studies for families identified in-person community-based methods as most effective [30]. However, in both of the aforementioned studies, the ultimate sample sizes were far smaller than our study. Although text blasts proved to be the most effective recruitment strategy, diverse recruitment methods using community engagement, where feasible, are still advised.

Even though community partners did not yield a high number of participants, our partnerships increased buy-in from participants, contributed to refining our research instruments, and helped us engage populations with less technology literacy and that are transient by providing feedback on survey front-end design and directly engaging with such groups. Other evaluations of SNAP and SNAP-Ed have similarly engaged with community partners to facilitate recruitment; however, none of these studies reported using remote recruiting strategies like text blasts [[32], [33], [34], [35]]. It was essential to balance the numerous security tactics employed in our study by building rapport and trust with both participants and community partners. Previous research has shown that some, particularly older adults and populations of lower income backgrounds, are distrustful of digital tools [[36], [37], [38]], which could introduce important participation barriers. Furthermore, the variability in the technological literacy of participants made it essential to engage community partners to facilitate in-person recruitment or assistance. We sent trained research assistants to community partner events with iPads to assist interested participants with the survey. In addition, trained research assistants aided participants over the phone. Although it is not possible to eliminate all trust or technology barriers to participation, the considerable efforts to increase the accessibility of this survey substantially reduced participation barriers [39].

Our data demonstrate that each security strategy has its own strengths and weaknesses. To best ensure data integrity, a combination of approaches should be used. First, with regards to survey design, single-use links and authentication offer a first layer of protection and allow for tracking and assessments of embedded data. Regarding specific security features, similar to other studies, we observed that basic, built-in features, specifically reCAPTCHA, can be effective and easy-to-use but are imperfect on their own [6,13,15,16]. For instance, in a study that recruited through Facebook and Twitter, all respondents, including suspected bots, passed reCAPTCHA [12], whereas another study that also recruited via social media found that 17.1% of responses failed reCAPTCHA [6]. In addition to reCAPTCHA, flagging duplicate IP addresses can be an effective and low-effort method to detect bots. Other studies have also found that duplicate IPs are indicative of bot behavior [12], with one study identifying nearly 600 duplicate IPs of over 2000 responses [13] and a recent public health study found 46.7% of suspected fraudulent actors had duplicate IPs [40]. However, tracking duplicate IP addresses may not be sufficiently precise when recruiting with community partners and/or allowing participants within the same household. As observed in our study, true participants sometimes completed the form multiple times and/or participants recruited at community partner sites often triggered duplicate IP addresses due to multiple interested individuals sharing the same Wi-Fi network. Although the majority of suspected bots had duplicate IP addresses, fraudulent actors may use virtual private networks (VPNs) to spoof their IP address and location [12]. VPNs can also be used by true participants—some of our community partners provide services to victims of domestic violence so they conceal building locations and likely use VPNs for privacy and security. Furthermore, the quality of duplicate IP addresses is dependent on the provider of such data. Dennis et al. [29] discuss how IP geolocations can be assigned to large swaths of people thus limiting the utility of precise geolocations and sometimes IP addresses. Although we observed similar challenges as Dennis et al. [29], duplicate IP addresses and geotags outside of the target recruitment areas were largely indicative of suspected bots in our sample.

We found that flexible and multipronged recruitment and security strategies were the most effective. For example, during high-text blasts periods, research assistants called suspected bots and random participants. As other studies have found [16,41], phone calls are a time-intensive but effective endeavor; however, we had allocated additional research assistant time during this period. Furthermore, when most recruitment occurred in-person and thus there was a lower risk of bots, research assistants were assigned to other tasks as random calls were unnecessary. Similarly, duplicate IP checks were ineffective while research assistants were recruiting participants in person at a community site where all participants, including the study’s iPads, were all on the same Wi-Fi network. When feasible, extended fielding timelines with ebbs and flows for recruiting can allow for proper resourcing and staffing. Although foundational security measures, such as reCAPTCHA, should always be present, more intensive measures (e.g., random phone calls) and/or less specific strategies (e.g., duplicate IPs) can be scaled up or down as needed to meet the needs of a particular study and most importantly paired with the appropriate recruiting tactics.

As others have documented [6,12,14,15,41], it is critical to include language in IRB applications and consent forms that documents the requirement for data quality checks to be met for incentive disbursement. Without such language, thousands of dollars may be spent on bot responses, as has unfortunately occurred in other studies [14]. Griffin et al. [6] noted how incentives as low as $5 can be lucrative for bots given the opportunity to repeatedly take a poorly secured survey. Given that our survey offered a $50 electronic gift card, there was a greater risk of fraudulent actors. As a result, our consent form explicitly stated that all responses would be reviewed by study staff and that if an issue was detected that a study team member would reach out to clarify any responses. By implementing automatic data checks and aforementioned security measures, we saved thousands of dollars in research funds that would have gone to fraudulent actors.

The use of VioScreen coupled with our automated data quality checks was efficient for both participants and researchers. Traditional paper FFQs cannot support complex skip patterns and rarely include photos of food which can help participants with recall [7,42]; these limitations are overcome with online FFQs which have demonstrated acceptable validity compared with traditional tools [43]. Online FFQs also offer cost savings to researchers compared with self-administered FFQs delivered via pen/paper self-administered or researcher-administered FFQs [7,44]. In our study, research assistants’ time for FFQs was limited to participants who failed data quality checks. There are still inevitable limitations of self-administered and online FFQs, for instance, repeated 24-h recalls yield more accurate estimates [7]. However, when conducting large-scale trials, like the What’s On Your Plate study, these concerns are outweighed by the cost and time savings offered by online FFQs which are necessary to recruit the sufficient sample size.

A key driver of our success was built on the study team’s ability to pivot toward integrating newer methods in collaboration with computer scientists. As the objective of the overall study is to understand the diet of SNAP recipients in RI and CT, we required a large sample size and a validated nutrition research tool. We worked with engineers at VioCare who assisted with the API endpoints that were crucial to setting up automated nutrition data quality checks. In addition, we hired computer science graduate students, with extensive experience and knowledge in API development, who were briefed on the objective of the study, the available resources, and the study needs. This involvement of computer scientists is a strength, unlike other studies that attempted bot mitigation efforts without such support [10]. Our security features were extremely cost effective as we worked with graduate-level computer scientists and used existing features within AWS and Qualtrics, which are supported by our respective institutions. Graduate-level students were recruited from our institutions and their support cost $20–$30 per hour with most of our APIs requiring 20–30 hours of consulting, development, and testing. Although our institutions support access AWS, each API call costs fractions of a cent [45]. As public health researchers, we became far more familiar with APIs and their respective endpoints, complex features of Qualtrics backend (e.g., workflows, API integrations, JSON events), and AWS to better understand our system and to debug and troubleshoot any issues.

In conclusion, our experience underscores how online surveys for public health nutrition research must be well protected from bots while balancing participant ease and access. As bots and fraudulent actors become more advanced, public health nutrition researchers should look to diversify their skills, research teams, and collaborations to include computer science. Leveraging existing infrastructure (e.g., Qualtrics, AWS), built-in features of web surveys (e.g., reCAPTCHA), and using graduate-level computer scientists can help reduce costs for smaller studies. Although this article highlights the best practices for our study, selected security approaches inevitably depend on specific study context. Nonetheless, based on our experience from the What’s On Your Plate study, we believe that public health research will increasingly require domain expertise, like computer science, that is not traditionally taught in health curricula. To complete high-quality research that leverages online data collection, it is important to have a cursory understanding of what bots are and how they work. Building in effective security features requires a careful balance of ensuring security by implementing measures that stop bots while minimizing barriers for participants of all digital literacy levels.

## Author contributions

The authors’ responsibilities were as follows – MKV, AT: designed the overall research; MKV, AT, EGE: led the data collection; EGE, JSS: created and designed technical backend; JSS: created the application program interfaces and receipt scraping end; EGE: analyzed the data and drafted the article; and all authors: interpreted the data, edited, read, and approved the final manuscript.

## Data availability

Data described in the manuscript will not be made available due to Institutional Review Boards requirements. Computer codes to develop application program interfaces will be provided by request.

## Funding

This work is funded by the U.S. Food Policy Evaluation Program at the University of Illinois Chicago, which is supported by a grant (2020-85774) from Bloomberg Philanthropies' Food Policy Program (www.bloomberg.org). MKV received funding from the K01 Career Development award from the National Heart, Lung, and Blood Institute (5K01HL165104).

## Conflict of interest

The authors do not have any conflicts of interest to disclose.
