# Supplementary material for: Population Recruitment Strategies in the Age of Bots: Insights from the What Is on Your Plate Study
Source: Curr Dev Nutr. 2025 Apr 15;9(5):107442. doi: 10.1016/j.cdnut.2025.107442 (PMC12143651; doi:10.1016/j.cdnut.2025.107442)

Population Recruitment Strategies in the Age of Bots: Insights from the What's on Your Plate Study

Emily G. Elenio

Supplemental Figure 1: Example Structure of Authenticated Link

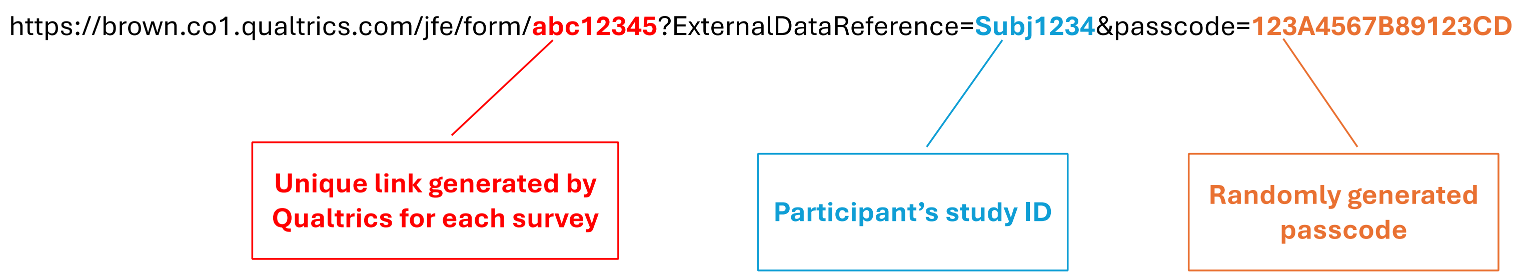

Supplement: Multimedia component 1 [file mmc1.pdf]
